# Perceptions of academic leaders in low- and middle-income countries about the role of WFME in enhancing the quality of medical education

**DOI:** 10.1371/journal.pgph.0005811

**Published:** 2026-01-08

**Authors:** James Kelly, Janet Grant, Mohammed Ahmed Rashid, Shanila Anwar, Muhammad Shahid Shamim

**Affiliations:** 1 Centre for International Medical Education Collaboration (CIMEC), University College London, United Kingdom; 2 The Centre for Medical Education in Context (CenMEDIC), United Kingdom; 3 The London School of Hygiene and Topical Medicine, United Kingdom; 4 The Aga Khan University, Karachi, Pakistan; University of Global Health Equity, RWANDA

## Abstract

Despite the significant proportion of medical schools located in low- and middle-income countries (LMICs), little is known about how educational leaders in these contexts perceive various roles of the World Federation for Medical Education (WFME) and its connection with the Educational Commission for Foreign Medical Graduates (ECFMG). This article aims to explore how the role of WFME is perceived by medical educators and accrediting body members in LMICs, including WFME Standards for Basic Medical Education (SBME), the recognition programme, and the challenges and motivations in adopting SBME and seeking WFME recognition. This qualitative study employed semi-structured interviews with ten senior medical educationalists and members of national accreditation bodies from Pakistan, Sri Lanka, the Philippines, and Indonesia. Thematic analysis was conducted using an inductive approach to identify key themes regarding perceptions of WFME as an organization, its recognition programme, and its standards. Eleven themes emerged from the analysis, the most prominent of which was a considerable conceptual uncertainty about WFME’s role and authority among participants. The relationship between WFME and ECFMG was frequently misunderstood, with recognition often viewed primarily as a mechanism to facilitate graduate mobility rather than to improve educational quality. While WFME standards were perceived as sufficiently broad to allow contextual adaptation, participants identified significant challenges including resource constraints and political pressures when implementing standards and pursuing recognition. This study demonstrates that the pursuit of WFME recognition by accreditation agencies in LMICs appears to be driven more by external pressures than by conviction about its intrinsic value for quality improvement. Greater clarity about WFME’s role, improved communication about the recognition process, and more explicit attention to the needs and contexts of LMICs could enhance the relevance and impact of these global medical education initiatives.

## Introduction

### Global standardization

The increasing globalization of medical practice has intensified the call for global quality assurance mechanisms. The increasing number of migrating doctors, cross-border education providers, and the proliferation of medical schools of varying quality, have accentuated the need to define standards and introduce effective and transparent accreditation systems [1]. This globalization has transformed medical education from a predominantly national concern into an international one.

The movement toward global standardization in medical education gained momentum with the establishment of the World Health Organization (WHO) and World Federation for Medical Education (WFME) Strategic Partnership in 2004 [2]. WFME is a non-statutory, not-for-profit, non-governmental organisation, established in 1972 [3]. It aims to promote the education and training of medical doctors worldwide [4]. Its objectives are to promote accreditation, to raise standards for basic and postgraduate medical education and continuing professional development through the publication of expert consensus of minimum and quality standards, and to maintain the World Directory of Medical Schools jointly with the Foundation for Advancement of International Medical Education and Research (FAIMER) [5]. WFME also has a close connection with the Educational Commission for Foreign Medical Graduates (ECFMG), which is the authorised testing agency for graduates of non-U.S. medical schools intending to apply for a U.S. medical residency programme [6].

The WHO-WFME partnership reflected recognition of the important interface between medical education and healthcare delivery, and the need for coordinated international efforts to improve educational quality. The WFME Global Standards program, initiated in 1997, has become the cornerstone of international efforts to harmonize medical education quality assurance [7]. These standards, developed through international taskforces involving more than 60 medical education experts from across the six WHO-WFME regions, represent a global expert consensus on minimum requirements and quality improvement benchmarks for medical education [8]. The partnership’s 2005 publication of guidelines for accreditation of basic medical education marked a significant milestone in the formalization of global quality assurance mechanisms. By 2011, WFME estimated that approximately half of medical schools worldwide had utilized these standards in some capacity [7].

The standards address eight universal themes: mission and values, curriculum, assessment, students, academic staff, educational resources, quality assurance, and governance and administration [7]. Their adoption of a principles-based approach in 2020 reflects recognition of the need for contextual adaptation while maintaining core quality principles [1]. This evolution acknowledges that while global standards can provide a framework for quality assurance, their implementation must be sensitive to local contexts, resources, and educational traditions.

### Global accreditation

Accreditation, defined by WFME as “certification of the suitability of medical education programs, and of competence in the delivery of medical education,” has emerged as the primary mechanism for operationalizing quality standards [9]. However, there is minimal empirical evidence demonstrating accreditation’s direct impact on educational quality or patient outcomes [10]. Recent research has begun to address this evidence gap, and suggests that accreditation processes may influence institutional quality improvement through their effects on governance, data collection and analysis, monitoring, documentation, and faculty engagement [11]. However, concerns have been raised about the costs, impacts on faculty morale, and potential for bureaucratization that may accompany accreditation processes [12].

### Critical perspectives

The global standardization movement has not been without critics. Scholars have raised significant questions about the assumptions underlying harmonized standards and their potential implications for educational diversity and local responsiveness. Rashid and Grant have highlighted concerns about the politicisation of global medical education, noting that “politics is characterized by power relations, and the deployment of power is inescapably political” [13]. This political dimension of standardization raises questions about whose values and priorities are embedded in global standards and how power dynamics influence their development and implementation.

As Whitehead has observed, there are significant concerns about the colonial dimensions of global medical education initiatives for which “the rationale is educational; the practice is colonial” [14]. This critique extends to accreditation systems, where global standards may inadvertently reinforce hierarchical relationships between Global North and Global South institutions [15].

The concept of academic colonialism has gained traction in discussions about global medical education governance [16]. This framework suggests that contemporary standardization efforts may reproduce historical patterns of domination, where Global North institutions and organizations establish norms and standards that Global South institutions are expected to adopt - often without meaningful participation in their development or adaptation to local contexts.

### The WFME recognition programme

The establishment of the WFME Recognition Programme (RP) in 2012 marked a significant expansion of global standardization efforts. Developed in response to the Educational Commission for Foreign Medical Graduates’ (ECFMG) 2010 policy requiring international medical graduates to graduate from appropriately accredited medical schools, the RP has evaluated and recognized accrediting bodies worldwide [6]. By 2022, WFME had recognized 33 accrediting bodies and received applications from another 16, encompassing over three-quarters of the world’s medical schools [17].

However, the rapid expansion of WFME’s influence has occurred “without significant awareness or scrutiny,” leading to calls for greater transparency, stakeholder engagement, and research evaluation [18]. The RP’s development was primarily driven by external policy requirements rather than evidence-based assessment of its potential to improve educational quality [18]. The RP has also been criticized for its potential to create perverse incentives, where institutions pursue recognition primarily for its external benefits (such as enabling graduate mobility) rather than for its intrinsic value in improving educational quality [19]. This instrumentalization of accreditation may undermine its intended purpose of quality improvement and create additional burdens for institutions in resource-constrained settings.

### Challenges in low- and middle-income countries

The medical education systems in low- and middle-income countries (LMICs) aim to prepare physicians to improve the healthcare of people in their own regions, and these regions often differ significantly from the Global North in the socio-cultural environment, healthcare services, educational needs and standards, and accreditation processes [20]. More than two-thirds of undergraduate medical institutes in the world are situated in these developing countries, and their graduates also make up a significant percentage of the healthcare workforce in Global North countries [21]. Many of these nations increasingly look to WFME for guidance and recognition [22].

However, the implementation of global standards in low- and middle-income countries (LMICs) faces significant challenges, [22] including resource constraints, limited expertise in quality assurance, and misalignment between global standards and local healthcare needs [22]. The principles-based approach adopted by WFME in 2020 was partly intended to address these concerns by allowing greater flexibility in implementation while maintaining core quality principles.

Nevertheless, the tension between global standardization and local contextualization remains a central challenge in medical education quality assurance. Critics argue that even principles-based standards may inadequately address the structural inequalities that characterize global medical education [13]. This perspective suggests that the challenge extends beyond technical implementation to fundamental questions about whose knowledge and priorities are privileged in global standardization efforts. While proponents argue that global standards can drive quality improvement and facilitate international mobility, critics contend that they may inadvertently undermine local innovation, cultural responsiveness, and educational diversity in a complex multipolar world [23].

### Frameworks for localization

The increasing drive for global standardization will, however, not inevitably lead to educational convergence. Dale’s exploration of the concept of a globally structured educational agenda suggests that global economic imperatives and supranational actors shape an agenda which is then mediated, reinterpreted, and localized by national states and other actors [24]. His work consistently emphasizes the complex interplay of global pressures and local agency, and how this produces significant local variation.

Similarly, Steiner-Khamsi’s Global-Local Politics Framework provides a critical lens for analysing policy transfer in education, exploring the complex political dynamics, strategic uses of external references, and active local reinterpretation that shape educational reforms driven by globalization [25]. The framework highlights that policy borrowing is not a neutral or technical process but a deeply political one: involving selective borrowing, based on existing domestic policy agendas or perceived needs, and legitimation, where external references are used to legitimize pre-existing policy preferences or to silence dissenting voices. Steiner-Khamsi’s work underscores the persistent diversity and unique local manifestations that arise from the interaction of global influences and local contexts. She emphasizes that even when policies appear similar on the surface, their meaning, implementation, and effects can vary significantly due to local reinterpretation.

## Research questions

Given the growing global influence of WFME in guiding standards and promoting quality in medical education, understanding its reception and perceived relevance in LMICs is critical. While standardization efforts have created frameworks for quality assurance and facilitated international cooperation, they have also raised important questions about power, participation, and the appropriateness of one-size-fits-all approaches to educational quality [12]. Understanding how these global initiatives are perceived and implemented in diverse contexts, particularly in LMICs, is essential for ensuring that quality assurance mechanisms serve their intended purpose of improving medical education and, ultimately, healthcare outcomes for all populations.

However, no studies to date have examined how the faculty and educational leaders in LMICs view WFME and its various roles. This is especially important due to the unique challenges faced by LMICs that may affect how global standards are interpreted and implemented [22].

Therefore, the primary research questions guiding this study are:

- How is the role of WFME perceived by medical educators and accrediting body members in LMICs?
- What are the beliefs about the purposes of the WFME Standards for Basic Medical Education (SBME) and recognition programme?
- What are the motivations and challenges in LMICs while adopting SBME and seeking WFME recognition?

The findings of this study will generate insights and help bridge the knowledge gap on how international frameworks align with local needs and inform future policy, adaptation strategies, and capacity-building efforts tailored to LMIC contexts. It will also contribute to global dialogue on equity and inclusiveness in health professions education reform.

## Methods

### Ethics statement

Ethical approval was obtained from The Aga Khan University Ethics Review Committee (8140–23955). Formal informed written consent to participate in this study was obtained from all participants, documented with e-signature and witnessed by the two interviewers.

### Research design

#### Theoretical framework.

**Fig 1:**
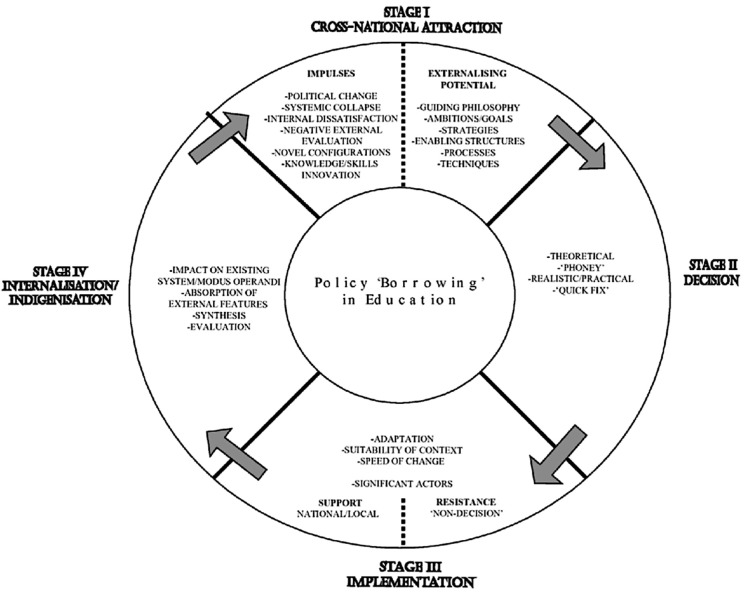
Policy ‘Borrowing’ in Education, from Philips & Ochs 2003 [26].

To explore the perceptions of academic leaders in LMICs about the role of WFME, this study employs a hybrid theoretical approach combining multiple complementary frameworks. The primary framework is the Composite Model developed for analysing policy ‘borrowing’ in the field of education [26], represented in Fig 1. This model views the process of change in education through four stages: (1) cross national attraction: impulses and externalizing potential (2) making a decision to change (3) implementation in the context of the borrowing country (4) internalization/ indigenization of the policy [27].

This framework is supplemented by Dale’s Localization Theory, which acknowledges the agency of local actors, here academic leaders, in modifying and integrating global standards [28]. Steiner-Khamsi’s Global-Local Politics of Policy framework informs the exploration of how WFME’s standards are used for legitimacy or resisted [25]. This hybrid approach provides a framework for understanding not only the adoption of WFME standards but also the underlying motivations, power dynamics, and local challenges involved in this process.

#### Conceptual framework.

**Fig 2:**
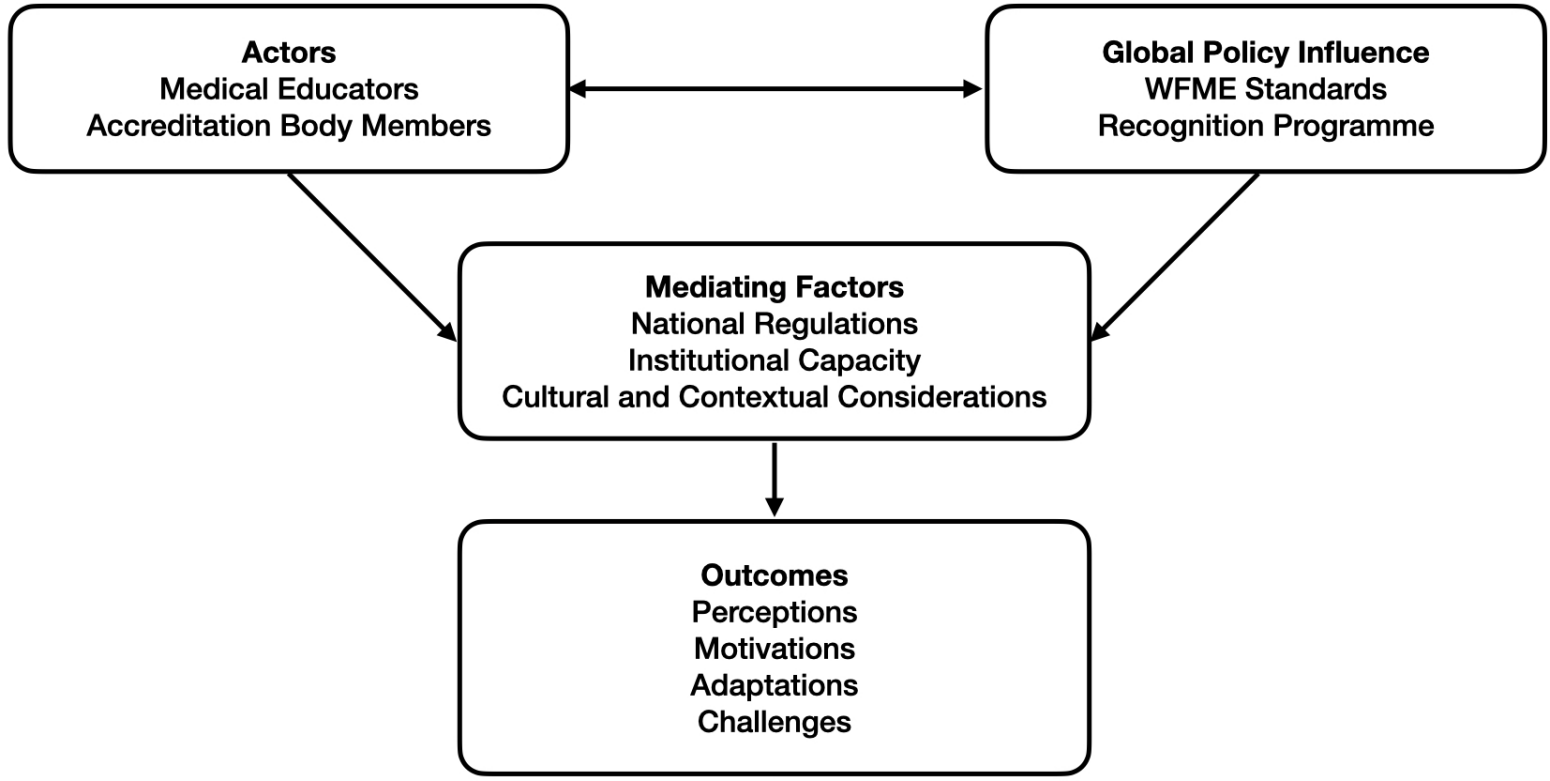
Conceptual Framework.

An exploratory qualitative approach was used for collecting data to understand interactions between actors (educators/accreditors), global policy (WFME), and local context (LMICs) leading to perceptions, motivations, adaptations, and challenges. This framework, which is represented in Fig 2, guided the study, allowing understanding of how WFME recognition and standards are understood, negotiated, and implemented in LMIC contexts.

### Sampling strategy and participants

Purposive sampling was employed to recruit participants who had direct experience with WFME standards and recognition processes. Direct experience was operationally defined as participants having led, participated in, or held responsibility for institutional activities specifically related to WFME standards adoption or recognition processes. This was assessed through initial screening questions about participants’ roles in WFME-related institutional processes and validated through follow-up questions during interviews about specific activities and responsibilities.

The inclusion criteria were: (1) senior medical educationalists with leadership roles in medical schools, (2) current or former members of national accreditation bodies, (3) individuals with direct experience of WFME standards implementation or recognition processes, and (4) ability to participate in English-language interviews. Exclusion criteria were: (1) those unable to provide informed consent (2) individuals engaged in dispute with their institutions regarding WFME standards and recognition processes.

Recruitment began on 1st March 2023 and ended on 31st March 2023, resulting in ten participants from four LMICs: Pakistan (4), Sri Lanka (2), the Philippines (2), and Indonesia (2).

### Data collection

Semi-structured interviews were conducted over video call in English by SA and MSS. The interview guide was developed based on the theoretical framework and research questions, covering topics including perceptions of WFME’s role, experiences with standards implementation, motivations for seeking recognition, challenges encountered, and views on the relationship between WFME and ECFMG. The interview guide was pilot tested with one participant to ensure clarity and appropriateness of questions.

Interviews were recorded with participant consent, transcribed verbatim, and anonymized to protect participant confidentiality. Interview duration ranged from 45-75 minutes, allowing for in-depth exploration of participants’ experiences and perspectives.

### Data analysis

Thematic analysis was conducted using an inductive approach. The analysis followed Braun and Clarke’s six-phase approach: familiarization with data, generating initial codes, searching for themes, reviewing themes, defining and naming themes, and producing the report [29].

Analysis was conducted collaboratively over a week-long workshop involving the research team. Coding rules were agreed upon among team members, and inductive coding was undertaken by two authors independently to enhance credibility. Initial codes were generated from the data, then grouped into potential themes. Themes were reviewed and refined through discussion among all authors to ensure they accurately represented the data and addressed the research questions.

While frequency of codes was noted to provide descriptive insight, the analysis prioritized thematic importance and contextual meaning over numerical frequency, recognizing that less frequent codes may offer deeper insights critical to understanding the research questions.

## Results

### Overview

Of the participants, 7 (70%) had served or were currently serving on their national accrediting body, 6 (60%) were professors of medicine or a closely related subject within a medical school, and 5 (50%) were male. This sample size was appropriate for an exploratory qualitative study aimed at understanding diverse perspectives rather than achieving statistical generalizability. Thematic analysis identified 139 codes, 35 categories, and 11 themes from the interview transcripts. These are reproduced in S1 Appendix, with quantification of codes.

### Theme 1: Conceptual uncertainty about WFME’s role

There was a considerable uncertainty about the role of WFME among participants. Four participants described it in general terms as a global organization with responsibility for medical education, for example as “the organisation that is really responsible for try[ing] to have better medical education across the world.” Three participants mentioned that its role primarily concerned standardisation, particularly through setting minimum standards for basic medical sciences. Three participants described it as a regulatory body, with only one correctly stating that it is not a regulatory body itself but rather aims to have oversight of regulatory bodies.

This uncertainty extended to WFME’s authority and legitimacy. The authority of WFME was linked by four participants to its association with other global and regional bodies. One described it as “an affiliate of the WHO,” and two drew connections to specific regional institutions from the Global North, suggesting that “Western associations, like AMEE, are informally part of the WFME.”

### Theme 2: Misunderstanding of relationship with ECFMG

The closest connection participants drew was to ECFMG, with five participants attributing the perceived influence of WFME to its link to ECFMG and its Recognition of Accrediting Agencies Policy (RAP) - “ultimately what has influenced the accrediting bodies is ECFMG.” However, the nature of this connection tended to be significantly overstated, with three participants erroneously expressing their belief that WFME recognition would exempt students from having to sit USMLE examinations.

One participant noted the complexity: “The ECFMG doesn’t accredit, it’s WFME and FAIMER [who] accredit [i.e., recognize] agencies for them.” This misunderstanding had significant implications for motivations to seek recognition.

### Theme 3: External pressures driving pursuit of recognition

Perceived motivations for accreditation agencies to seek WFME recognition centred on the benefit to students of being able to work in the US and the consequent marketability of medical schools which had been accredited by a recognised agency. Comments about this outweighed comments about potential improvement in standards.

This close connection with ECFMG was seen as a substantial contributor to the sense that seeking WFME recognition had essentially become compulsory. Nine participants expressed a feeling that graduate opportunities to work abroad would be severely limited if WFME recognition were not gained, with one stating “if we don’t submit to accreditation [meaning recognition] then we don’t get to send students abroad.”

One participant described this as a loss of autonomy: “the decision by ECFMG [took] away a lot of our autonomy - we really had no choice, and we had to comply.” This impression was reinforced by participants who stated that “disadvantageous or not, we’re still going to be doing it.”

### Theme 4: Standards as quality improvement mechanism

For those who identified positive benefits of recognition, statements centred on the way standards could raise quality. One participant observed of medical schools that “definitely their teaching and learning processes will be much better.” However, the mechanism by which this might happen was not consistently identified, with some pointing to the systematisation of local standards, the broader benefits of reflecting on local standards while adapting them to SBME, and the benefits of the move to qualitative from quantitative assessment.

### Theme 5: Contextualisation challenges and opportunities

There was general agreement that standards were broad and non-prescriptive, particularly in their latest iteration, with one participant commenting that “there is a lot of latitude - they are not binding.” Contextualising WFME standards was widely seen as unproblematic, largely due to their broad and non-specific nature. As one participant noted, “[standards] can easily be customized since the basic medical education criteria are so broad.”

However, this breadth sometimes created uncertainty about compliance requirements and authentic indigenization that would make standards genuinely reflective of local healthcare needs and educational traditions.

### Theme 6: Resource constraints and process burdens

Issues around compliance with recognition processes centred on the expense, both in terms of money and time required. As one participant noted, “it’s very expensive and time-consuming.” These resource constraints were particularly challenging for institutions in resource-limited settings, creating implementation disparities between better-resourced and less-resourced institutions.

### Theme 7: Commercialisation concerns

The recognition process was increasingly viewed through a commercial lens. One participant described it as having “become more of a commercialised venture than an academic exercise where reviewers and the different institutions share experience.” This commercialisation was seen as detracting from the educational value of the process.

### Theme 8: Political pressures

Political pressures emerged as significant factors in decision-making processes. Three participants specifically identified ministerial intervention, with one noting that “the minister of education and the minister of health have tried... to push through” recognition efforts. This suggested that decisions were often made at high political levels rather than through grassroots educational consensus.

### Theme 9: Fear of marginalisation

The pursuit of recognition was driven partly by fear of being left behind. One participant noted that recognition “has become normative,” creating pressure to conform regardless of perceived value. This fear of marginalisation was expressed as “disadvantageous or not, we’re still going to be doing it.”

### Theme 10: Reputational considerations

Recognition was increasingly viewed as “a hierarchical endorsement exercise” rather than a meaningful quality improvement process. This hierarchical framing created additional pressure to pursue recognition for reputational rather than educational reasons.

### Theme 11: Postcolonial perspectives

Across all participants, there appeared to be a perception that the ECFMG RAP and the WFME recognition programme represent the imposition of a policy by the Global North on the Global South. For some, this perception was expressed in postcolonial terms, with one participant saying, “it is the colonial mindset that is still working.”

## Discussion

### Cross national attraction: Impulses and externalizing potential

The attraction of WFME standards and recognition among LMICs appears multifactorial, comprising complex external and internal factors that extend beyond intrinsic quality improvement motivations. The most powerful external impulse identified by participants was the connection between WFME recognition and ECFMG’s RAP. This powerful externalizing potential of WFME recognition - the ability to facilitate graduate mobility to the United States - overshadowed considerations of its intrinsic value for quality improvement.

The reputational dimension of cross-national attraction also emerged as significant, with participants describing the hierarchical nature of global medical education. This hierarchical framing created a fear of marginalization among those who might not pursue recognition.

Notably absent from most participants’ accounts were impulses related to improving local healthcare delivery or addressing specific educational challenges within their contexts. The cross-national attraction appeared predominantly oriented toward external validation and global positioning rather than addressing internal needs.

This may have profound implications for the authenticity of quality improvement initiatives in LMIC medical education. The pursuit of standards primarily to facilitate graduate emigration rather than to enhance local healthcare capacity may exacerbate existing “brain drain” challenges while also failing to address fundamental issues of educational quality. Participants who explicitly attributed WFME’s influence to its ECFMG connection may be identifying that that medical education policy in LMICs is increasingly driven by Global North labour market demands rather than local healthcare needs.

### Making a decision to change

The decision-making processes regarding adoption of WFME standards and pursuit of recognition were characterized by multiple, sometimes competing, influences. Political pressures emerged as significant factors, with ministerial intervention often driving decisions at high political levels rather than through grassroots educational consensus.

Student and market pressures also heavily influenced decision-making, with participants describing significant demand from students for international mobility opportunities. This commercial dimension was explicitly acknowledged by participants.

Decision-making was further complicated by conceptual uncertainty about WFME itself. The varied and sometimes contradictory understandings of WFME’s role and authority among participants suggest that decisions were often made without complete information about what was being adopted and why.

These decision-making patterns have significant implications for the sustainability and effectiveness of medical education reforms in LMICs. When changes are imposed rather than chosen, institutional commitment to implementation and continuous improvement may be compromised, potentially leading to superficial compliance rather than genuine change.

### Implementation in the context of the borrowing country

The implementation of WFME standards and recognition processes in LMIC contexts revealed significant adaptation challenges. Resource constraints emerged as a primary implementation barrier, with participants consistently highlighting the financial and time burden of the recognition process.

Beyond financial resources, participants identified human resource and expertise limitations as implementation challenges. The technical requirements of standards implementation and recognition processes often assumed administrative and educational expertise that was not uniformly available across all settings.

Some participants framed implementation challenges in postcolonial terms, suggesting that implementation was sometimes experienced as an imposition of external values rather than a collaborative process of quality improvement.

Despite these challenges, participants’ descriptions of contextualizing implementation through selective adaptation and local interpretation suggest that meaningful change may still be possible when standards are sufficiently flexible. However, this contextual adaptation requires significant local capacity that may not be uniformly available, again raising equity concerns about who benefits from global standardization efforts.

### Internalization and indigenization

The internalization and indigenization of WFME standards and accreditation processes in LMICs emerged as an ongoing and uneven process. While some participants had internalized the value of standards for quality improvement, others expressed a more instrumental approach, viewing them primarily as external requirements to be met rather than values to be internalized.

The emphasis on international compatibility, particularly for enabling graduate mobility, created pressure for conformity that could potentially undermine authentic indigenization. This tension was reflected in the description of standards implementation as sometimes becoming “more of a hierarchical endorsement exercise” rather than a meaningful engagement with local educational values and priorities.

These descriptions suggest that international organizations may need to fundamentally reconsider their communication and approach to ensure that standardization efforts genuinely serve local educational improvement. There may be a benefit from greater clarity and differentiation between WFME’s standard-setting and recognition functions, with more explicit attention to supporting authentic contextualisation rather than mere compliance. This might involve developing more collaborative approaches that engage LMIC institutions as partners in standards development.

### Implications for global medical education policy

These findings have several important implications for global medical education policy and practice. First, they highlight the need for greater clarity about WFME’s role and the distinction between its standard-setting and recognition functions. Second, they suggest that current approaches to global standardization may inadvertently prioritize external validation over local quality improvement needs.

The study also reveals the unintended consequences of linking recognition processes to graduate mobility, which may distort the primary purpose of quality assurance. Finally, the findings suggest a need for more culturally responsive approaches to global standard-setting that better account for the diverse contexts and needs of LMICs.

## Strengths and limitations

### Strengths

To the best of the authors’ knowledge, this study represents the first systematic exploration of how medical education leaders in LMICs perceive WFME, its standards, and recognition programme. By focusing specifically on LMICs, which host more than two-thirds of undergraduate medical institutes globally, the study addresses a significant gap in understanding how international quality assurance mechanisms are received in contexts that differ substantially from the Global North.

The multi-country approach, incorporating perspectives from four different LMICs across two regions, allowed for identification of common themes that transcend specific national contexts, enhancing the contextual understanding and depth of insights rather than statistical generalizability. The inclusion of participants with diverse roles - both those within medical schools and those serving on national accreditation bodies - provided complementary perspectives that enriched the analysis.

The methodology was appropriate for this exploratory study, with thematic analysis allowing for deep understanding of participants’ experiences and perspectives. The collaborative analysis process, with independent coding by multiple researchers followed by consensus discussions, enhanced the credibility and trustworthiness of the findings.

### Limitations

The sample was limited to ten participants across four Asian countries, which may not capture the full diversity of LMIC perspectives globally. Significant contextual differences found in sub-Saharan Africa, Latin America, or conflict-affected states are absent, meaning that findings may reflect regional patterns more than global LMIC realities. Future research should include broader geographic representation to capture more diverse LMIC contexts.

The study relied on self-reported perceptions and did not include objective measures of how WFME standards and recognition processes have been implemented in practice. This limits the ability to distinguish between perception and reality regarding the impact of WFME initiatives.

Some national agencies achieved WFME recognition after data collection. For example, the Pakistan Medical and Dental Council achieved recognition in 2024. This study did not examine whether perceptions of WFME recognition had changed since achievement of recognition. This would have provided interesting additional information.

All interviews were conducted in English, which may have limited the nuance and depth of expression for participants from non-English speaking countries, potentially affecting the richness of data collected. Additionally, the position of the researchers, some of whom may have prior connections to medical education systems in the studied countries, could have influenced both data collection and interpretation.

The study has not sought the perspectives from WFME representatives or from medical education stakeholders in high-income countries, since this is outside of its scope. However, this would have provided important contextual information for interpreting the perceptions identified and would be worthy of future research.

While the sample size was appropriate for an exploratory qualitative study focused on depth and contextual understanding, future research with larger and more geographically diverse samples would strengthen the evidence base and provide insights into regional variations in perceptions and experiences.

## Conclusion

Despite decades of investment in global standardization efforts, the evidence base supporting their effectiveness remains limited. Studies examining the impact of accreditation on educational quality have produced mixed results, with some suggesting positive effects on institutional processes while others questioning the relationship between accreditation and meaningful quality improvement [11,30]. The lack of robust evidence is partly attributable to the methodological challenges inherent in evaluating complex educational interventions and the difficulty of establishing causal relationships between accreditation and long-term outcomes.

Recent calls for evidence-based approaches to medical education governance have highlighted the need for more rigorous evaluation of standardization initiatives [31]. This includes not only assessment of their effectiveness in improving educational quality but also examination of their unintended consequences, differential impacts across contexts, and alignment with local healthcare needs and priorities.

The future of global medical education quality assurance will likely require navigating the tension between the benefits of international coordination and the need for local responsiveness [12]. This may involve developing more nuanced approaches that combine global principles with contextual adaptation, ensuring meaningful participation of diverse stakeholders in standard-setting processes, and investing in robust research to evaluate the effectiveness of different approaches to quality assurance.

This study reveals significant conceptual uncertainty about WFME’s role and the relationship between its standard-setting and recognition functions among academic leaders in LMICs. The findings suggest that the pursuit of WFME recognition is driven more by external pressures, particularly the connection to ECFMG’s RAP, than by conviction about its intrinsic value for quality improvement.

The research demonstrates the need for greater clarity about WFME’s role, improved communication about the recognition process, and more explicit attention to the needs and contexts of LMICs. The findings also highlight the importance of distinguishing between global standardization for external validation and quality improvement for local healthcare needs.

Future research should explore these themes across broader geographic regions and include perspectives from multiple stakeholders, including WFME representatives and medical education leaders from high-income countries. This would provide a more comprehensive understanding of how global medical education quality assurance mechanisms can be made more responsive to diverse contexts while maintaining their effectiveness in promoting quality improvement.

## Supporting information

S1 Appendix Codes, Categories and Themes. (DOCX)
