# Supplementary material for: Perceptions of academic leaders in low- and middle-income countries about the role of WFME in enhancing the quality of medical education
Source: PLOS Glob Public Health. 2026 Jan 8;6(1):e0005811. doi: 10.1371/journal.pgph.0005811 (PMC12782367; doi:10.1371/journal.pgph.0005811)
Supplement: S1 Appendix — (DOCX) [file pgph.0005811.s001.docx]

S1 Appendix: Codes, Categories and Themes

| **Standards as Quality Improvement Mechanism** | | | | | | | | | | | | |
| --- | --- | --- | --- | --- | --- | --- | --- | --- | --- | --- | --- | --- |
| Category | Code | Quotation | 1 | 2 | 3 | 4 | 5 | 6 | 7 | 8 | 9 | 10 |
| Uncertainty | uncertainty about alignment between local standards and WFME standards |  | x |  |  |  |  |  |  | x |  |  |
|  | uncertainty about benefits of recognition | "what benefit do we get from … having the WFME rubber stamp on it" (8) |  |  | x | x | x |  |  | x |  |  |
| Link between meeting standards and gaining recognition | accreditation agencies use WFME standards |  | x | x |  | x | x | x | x |  | x | x |
|  | recognition firmly linked to standards | if we meet their standards, they definitely give recognition to the regulatory body (2) |  | x |  |  | x |  | x |  | x | x |
|  | recognition not robust | when you go to the WFME list of institutions that have been recognized, you will see a lot of mist. That of institutions that actually should not have been recognized. (4) |  |  |  | x |  |  |  |  |  |  |
|  | no criteria given for length of recognition | where are the criteria? (3) |  |  | x |  |  |  |  |  |  |  |
|  | national standards are more specific and rigorous | that is important because that basically helps you to make sure that the clinical training is provided in the most rigorous way (5) |  |  |  |  | x |  |  |  |  |  |

| **Misunderstanding of Relationship with ECFMG** | | | | | | | | | | | | |
| --- | --- | --- | --- | --- | --- | --- | --- | --- | --- | --- | --- | --- |
| Category | Code | Quotation | 1 | 2 | 3 | 4 | 5 | 6 | 7 | 8 | 9 | 10 |
| Motivations & pressures | ECFMG RAP main motivator for seeking recognition | the entire story started with the ECFMG regulation (4) |  | x | x | x |  |  |  |  |  |  |
|  | ECFMG exerted pressure on WFME | ultimately what has influenced the accrediting bodies is ECFMG (4) |  |  | x | x |  |  |  |  |  |  |
|  | ECFMG action not based on educational principles | only the schools recognized by the accrediting bodies would be there in the would be allowed to appear in ECFMG; that was something that, was not very I didn't find it very educationally useful (3) |  |  | x |  |  |  |  |  |  |  |
|  | part of colonial mindset | it is the colonial mindset that is still working (3) |  |  | x |  |  |  |  |  |  |  |
| Benefits of recognition | recognition allows students to go to US | if they have a letter from our … accreditation body they will accept [that] … this student is a really [a] comparable fit' (7)  most of our doctors they want to move to USA . So that is the reason that our regulatory body would want to be recognized by WFME (2) | x | x | x |  |  | x | x |  |  | x |
|  | aligned primarily to US standards | they recognise … some countries follow US model and some … European model and … take into account' (7) |  | x | x |  | x |  | x |  |  |  |
|  | no consequences to not being recognised |  |  |  |  | x |  |  |  |  | x |  |
|  | recognition applies beyond US |  | x |  |  |  |  |  |  |  | x |  |
|  | recognition allows students to go to Europe | by using the recognition from the world federation of medical education they accept a student permanently … to have the education in those areas (7) | x |  |  |  |  |  | x |  |  |  |
|  | sits alongside other accreditation processes |  |  |  |  |  |  |  |  |  | x |  |
| Uncertainty & complication | complicated link between WFME recognition for USMLE | It's very complicated … The ECFMG doesn't accredit, it's WFME and FAIMER [who] accredit [recognise] agencies [for] them (9) |  | x | x | x | x |  |  | x | x |  |
|  | uncertainty if recognition bypasses USMLE | I don't know … if not, what is it for?' (6) |  |  |  |  |  | x | x |  |  |  |
| Enforcement of RAP | unsure about enforcement | there is no need to follow it now (4) |  |  | x | x |  |  |  |  | x |  |
|  | enforcement delayed | "well that's good news …" (9) |  |  | x | x |  |  |  |  |  |  |

| **Standards as Quality Improvement Mechanism** | | | | | | | | | | | | |
| --- | --- | --- | --- | --- | --- | --- | --- | --- | --- | --- | --- | --- |
| Category | Code | Quotation | 1 | 2 | 3 | 4 | 5 | 6 | 7 | 8 | 9 | 10 |
| Benefits | way to raise quality | definitely their teaching-learning processes will be much better (2) |  | x |  |  |  | x | x | x | x | x |
|  | making national bodies standards more systematic |  |  |  |  |  |  |  |  |  | x |  |
|  | can improve systems and governance |  |  |  |  |  |  |  |  |  | x |  |
|  | fill local gaps in standards |  |  |  |  |  |  |  |  |  | x |  |
|  | multiple local, national and international standards provide checks and balances | there would be a lot of checks and balances (5) |  |  |  |  | x |  |  |  |  |  |
|  | opportunity for reflection | There were a lot of things that we had to provide to them (for recognition) and while providing .. it was an opportunity for us to reflect on how well we were performing our task as a regulatory body. (1) | x |  |  |  |  |  |  |  |  |  |
|  | confidence improved | our self-confidence will be improved in quality of our education (6) |  |  |  |  |  | x |  |  |  |  |
| Uses | can be used to produce inspection forms | we converted those standards into inspection forms (4) |  |  |  | x |  |  |  |  |  |  |
|  | as a starting point for national standards | in the beginning they were [aligned] but now we have gone beyond' (5) |  |  |  |  | x |  |  |  |  |  |
|  | benchmarking | we need to be at par with countries with maybe more advanced medical education systems (8) | x |  |  |  |  |  |  | x |  |  |
|  | can be used to generate competencies |  |  |  |  |  |  | x |  |  | x |  |
|  | practice can be aligned to tripartite standards |  |  |  |  |  |  |  |  |  | x |  |
|  | maintenance of an ingroup | you want to be on "the list" (9) |  |  |  |  |  |  |  |  | x |  |
| Nature | not specific to specialty |  | x |  |  |  | x |  |  |  | x |  |
|  | very broad standards | that's ok because if you are thinking about the global level accreditation, you can't go into very specifics' (5) | x |  | x |  | x |  |  |  |  | x |
|  | not prescriptive | they are not prescriptive, just suggestions … there is a lot of latitude - they are not binding (4) |  |  | x | x | x |  |  |  | x |  |
|  | evidence-based |  | x |  |  |  |  |  |  |  |  |  |
|  | compulsory | provides guidance and standards which graduates from different countries have to follow | x |  |  |  |  |  |  |  |  |  |
|  | related to WHO policy to have comparable standards between countries | [WFME standards] are related to the WHO policy that we have to have a comparable standard with other countries (7) |  |  |  |  |  |  | x |  |  |  |
| Process | qualitative rather than quantitative method | now we try to adjust our own standards to the qualitative method not the quantitative method (7) |  |  |  | x |  |  | x |  |  |  |
|  | just about process not about quality | they were just checking about the process that has been followed (5) |  |  |  | x | x |  |  |  |  |  |
| Adoption | pressure rather than obligation | we (are not) asked to follow them, but we're expected to follow them. So we don't really have any obligation, but there's a lot of pressure in terms of expectations that we have to follow them. (1) | x |  |  |  |  |  |  |  |  |  |
|  | no pressure to adopt international standards | We have not adopted … as we have not received requests to adopt international standards (6) |  |  |  |  |  | x |  |  |  |  |
| Relationship with national standards | standards are linked with national standards | when we try to match global standards with national standards [they become] really linked' (7) |  |  | x |  |  |  | x |  |  |  |
|  | higher standards than national standards | we achieve the national standard and then we plan to have the international standard' (7) |  |  |  |  | x |  | x | x |  |  |
|  | permission to adopt needed by national bodies | We were not allowed to use the standards … yet (6) |  |  |  |  |  | x |  |  |  |  |
|  | relies on accrediting bodies communicating standards well | blind faith …' (9) | x |  |  |  |  |  |  |  | x |  |
|  | no national conversation about WFME standards |  | x |  |  |  |  |  |  | x |  |  |
| Iterations | uncertainty about different iterations of standards | I think … yes … we use 2016 standards' (7) | x |  |  |  |  |  | x |  |  |  |
|  | 2015 standards seen as more prescriptive | the 2015 document was thought to be prescriptive and hence not usable by many countries. So they become less prescriptive (4) |  |  |  | x |  |  |  |  |  |  |
|  | 2015 easier to use | the 2015 document was still more looked at because it was more structured (4) |  |  |  | x |  |  |  |  |  |  |

| **Conceptual Uncertainty About WFME's Role** | | | | | | | | | | | | |
| --- | --- | --- | --- | --- | --- | --- | --- | --- | --- | --- | --- | --- |
| Category | Code | Quotation | 1 | 2 | 3 | 4 | 5 | 6 | 7 | 8 | 9 | 10 |
| Purpose | ensures coherence across regulatory regimes |  |  |  |  |  | x |  |  | x |  |  |
|  | needs to be global oversight of medical schools |  |  |  |  |  |  |  |  |  | x |  |
|  | has role throughout lifecycle - undergrad, postgrad, CPD |  |  |  |  |  |  |  |  |  | x |  |
|  | lead international advocacy about med ed |  |  |  |  |  |  |  |  | x |  |  |
|  | promote social relevance and responsiveness of med ed |  |  |  |  |  |  |  |  | x |  |  |
|  | making med ed more fit for purpose |  |  |  |  |  |  |  |  | x |  |  |
|  | can facilitate global community of research |  |  |  |  |  |  |  |  | x |  |  |
|  | responsibility for global medical education | This is the organisation that is really responsible for try[ing] to have better medical education across the world' (7) |  |  |  |  |  |  | x |  |  | x |
|  | monitoring progress | the WFME always monitor to ask about the progress (7) |  |  |  |  |  |  | x |  |  |  |
|  | setting minimum standards | it has played an important role in setting … minimum criteria for basic medical sciences (5) | x |  |  |  | x |  |  |  |  | x |
|  | introducing the concept of global standards | Its main purpose is to bring about standardization in medical education and to improve the quality of education around the world (1) | x |  |  |  | x |  |  |  |  | x |
|  | oversight of regulatory bodies | It offers oversight over many of the regulatory bodies that are working | x |  |  |  |  |  |  |  |  |  |
|  | to ensure standardisation | Its main purpose is to bring about standardization in medical education and to improve the quality of education around the world | x |  |  |  |  |  |  |  |  |  |
|  | good principle to establish recognition of accreditation | I would see this as a positive thing (5) |  |  |  |  | x |  |  |  |  |  |
|  | maintaining database |  |  |  |  |  |  |  |  |  | x |  |
| Nature | not a regulatory body | the WFME is not a regulatory body (2) |  | x |  |  |  |  |  |  |  |  |
|  | recognises accreditation agencies not schools |  |  |  |  |  |  |  |  |  | x |  |
|  | global organisation |  | x |  | x |  |  |  | x | x |  |  |
| Uncertainty | unsure of regulatory role |  |  |  |  | x |  | x |  | x |  |  |
|  | uncertainty about relationship with individual medical schools |  |  |  |  |  |  |  |  | x |  |  |
|  | unsure if focus is on high income countries |  |  |  |  |  |  |  |  | x |  |  |
| Relationships with other organisations | WFME and FAIMER accredit together |  |  |  |  |  |  |  |  |  | x |  |
|  | link to IFMSA |  |  |  |  |  |  |  |  | x |  |  |
|  | link to WHO | the WFME since its inception … was considered like an affiliate of the WHO (5) |  |  |  |  | x |  |  |  |  |  |
|  | Link to Western associations | Other Western associations, like AMEE, are informally part of WFME (5) |  |  |  |  | x |  |  |  |  | x |
| Reputation | the power of the name | the WFME name is powerful is making us realise we need quality in the education of medical students (6) |  |  |  |  |  | x |  |  |  |  |
|  | small and insubstantial | That there's no full-time worker, there's there are no offices. There are no technical experts to support gathering data to analyze that work (4) |  |  |  | x |  |  |  |  |  |  |
|  | not data driven | there is no data that has been made public - we don't know (4) |  |  |  | x |  |  |  |  |  |  |
|  | national conversation about national regulation more than WFME |  |  |  |  |  |  |  |  | x |  |  |

| **Contextualisation Challenges and Opportunities** | | | | | | | | | | | | |
| --- | --- | --- | --- | --- | --- | --- | --- | --- | --- | --- | --- | --- |
| Category | Code | Quotation | 1 | 2 | 3 | 4 | 5 | 6 | 7 | 8 | 9 | 10 |
| Role of accrediting bodies | accreditation agency contextualises standards |  | x |  |  |  |  |  |  |  | x |  |
|  | quality improvement cannot come from external agency | No international agency can help us improve (3) |  |  | x |  |  |  |  |  |  |  |
|  | no explicit mention in national standards about alignment with WFME |  |  |  |  |  |  |  |  | x |  |  |
|  | adaptation must be done locally | The Colombo medical school is now 150 years old. I suppose there are several medical schools in Pakistan with a very long tradition and established history ... so a very well-established tradition related to medical education and ... quality assurance and accreditation. | x |  |  |  | x |  |  |  |  |  |
|  | builds on rich history of quality assurance | there has been a very well-established tradition related to medical education and with that the quality assurance and accreditation goes in line …' (5) |  |  |  |  | x |  |  |  |  |  |
| LMIC | practice very different in developing world |  |  |  |  |  |  | x |  |  | x |  |
|  | does not disadvantage developing contexts | it would be unfair to all the efforts medical educators do in developing settings (9) |  |  |  |  |  |  |  | x | x |  |
|  | need for wide representation | they really need to involve representatives from low- and middle-income countries while they're framing those standards (1) | x |  |  |  |  |  |  |  |  |  |
|  | unfamiliar terminology | we build on British traditions … [in which] there is no mention of accreditation (5) |  |  |  |  | x |  |  |  |  |  |
| Process | not difficult to contextualise standards | it can easily be customised since the basic medical education criteria are so broad (5) |  |  |  |  |  | x | x |  | x |  |
|  | beginning with copying across | there was a bit of copy-paste' (4) |  |  |  | x |  |  |  |  |  |  |
|  | no need for contextualisation | they're not asking us to follow anything, they're just guidelines (3) |  |  | x |  |  |  |  |  |  |  |
|  | easier to adapt standards than write own | I am sure we have people in our country who can definitely sit together and do the job (write our own standards), but it's easier to take it from someone else (2) |  | x |  |  | x |  |  |  |  |  |

| **Commercialisation Concerns** | | | | | | | | | | | | |
| --- | --- | --- | --- | --- | --- | --- | --- | --- | --- | --- | --- | --- |
| Category | Code | Quotation | 1 | 2 | 3 | 4 | 5 | 6 | 7 | 8 | 9 | 10 |
| Marketability | accreditation by recognised agency is good for marketing / recruitment |  |  |  |  |  |  |  |  |  | x | x |
|  | market aspects: may need to move accrediting agencies to one recognised |  |  |  |  |  |  |  |  |  | x |  |
|  | longer accreditation more marketable |  |  |  |  |  |  |  |  |  | x |  |
|  | number of graduates going to US is marketable | it's sort of an honor for the institute (2) | x | x |  |  |  |  |  |  |  |  |
|  | marketing can sit USMLE |  |  |  |  |  |  |  |  |  | x | x |
|  | accreditation bodies not mentioning / advertising WFME connections |  |  |  |  |  |  |  |  | x | x |  |
|  | WFME low profile | We don't actually reference WFME in any of our communications because the general public probably doesn't know that the WFME actually exist' (9) |  |  |  |  |  |  |  | x | x |  |
| Wider market | requests from other countries to extend accreditation | We also received the request from the Korean and from the Russian country to have accreditation for medical education … and also from our neighbour … Malaysia (7) |  |  |  |  |  | x | x |  |  |  |
|  | vision to be a global accreditor | We are requesting permission to accredit … outside our jurisdiction' (6) |  |  |  |  |  | x | x |  |  |  |
| Process of commercialisation | perception that accreditation is commercialised | it has become more of a commercialised venture than an academic exercise where reviewers and the different institutions share experience (5) |  |  | x | x | x |  |  |  |  |  |
|  | need to return to original principles | accreditation is a good thing but there is a need to return to its original values and morals (5) |  |  |  |  | x |  |  |  |  |  |

| **Resource Constraints and Process Burdens** | | | | | | | | | | | | |
| --- | --- | --- | --- | --- | --- | --- | --- | --- | --- | --- | --- | --- |
| Category | Code | Quotation | 1 | 2 | 3 | 4 | 5 | 6 | 7 | 8 | 9 | 10 |
| Unproblematic | no issues in adapting standards |  |  |  |  | x |  | x | x |  |  | x |
|  | easy to get information directly | we always want the [primary] source for information' (6) |  |  |  |  |  | x |  |  |  |  |
|  | challenges at local level not at international level | the challenges were actually at the local level (5) |  |  |  |  | x |  |  |  |  |  |
|  | thorough | its extremely thorough (2) |  | x |  |  |  |  |  |  |  |  |
| Problematic | too many visits | over and over again (5) |  |  |  |  | x |  |  |  |  |  |
|  | WFME recognition is not concerned with the quality of medical education | they haven't gone into the quality of medical education (5) |  |  |  |  | x |  |  |  |  |  |
|  | Lack of clarity in recognition process | We could have been given more detail instructions regarding what they expect us to do (1) | x |  |  |  |  |  |  |  |  |  |
|  | Unresponsive | only source of contact that we had with them was via e-mail and didn't really receive very prompt replies (1) | x |  |  |  |  |  |  |  |  |  |
|  | Lack of feedback | There needs to be more discussion on that and they need to be more welcoming about feedback and they also need to take us on board while dropping those standards. (1) | x |  |  |  |  |  |  |  |  |  |
|  | Taking away autonomy | the decision by ECFMG that take(took) away a lot of our autonomy. We really had no choice and we had to comply. I did feel neglected I did feel powerless in that situation. (1) | x |  |  |  |  |  |  |  |  |  |
|  | time and opportunity costs | it's very expensive and time consuming (5) |  | x | x | x | x |  | x |  | x | x |
|  | unreasonable costs | they will come in business class, they will not come in economy (4) | x |  |  | x |  |  |  |  |  |  |

| **Postcolonial Perspectives** | | | | | | | | | | | | |
| --- | --- | --- | --- | --- | --- | --- | --- | --- | --- | --- | --- | --- |
| Category | Code | Quotation | 1 | 2 | 3 | 4 | 5 | 6 | 7 | 8 | 9 | 10 |
| Student needs | pressure from students to accredit to facilitate emigration | If we don't submit to accreditation [recognition]  then we don't get to send students abroad (9) |  | x | x | x |  |  |  | x | x |  |
|  | help to send students abroad | We need to make our workers more "export ready" (8) |  |  |  |  |  |  |  | x | x | x |
|  | breaks down barriers to work globally |  |  | x |  |  |  | x | x | x |  | x |
| Context | situated as part of wider labour export phenomenon | We are also preparing doctors for global export (8) | x |  |  |  |  |  |  | x |  |  |
|  | migration more related to economic factors than accreditation | so, all these things are nothing to do with the accreditation or minimum standards (5) | x |  |  |  | x |  |  |  |  |  |
| Quality assurance | WFME quality ensures workforce | Many of my colleagues [may think] that the WFME stamp is the seal of quality assurance (8) |  |  |  |  |  |  |  | x |  | x |
|  | quality improvement weighed against emigration | it's my way of reconciling this …' (9) |  |  |  |  |  |  |  | x | x |  |
|  | producing globally competitive workers | We are in a bipolar position ... a push and pull (8) |  |  |  |  |  | x | x | x |  |  |
| Ethics | "fairness" not an issue |  |  |  |  |  |  |  |  |  | x |  |

| **Reputational Considerations** | | | | | | | | | | | | |
| --- | --- | --- | --- | --- | --- | --- | --- | --- | --- | --- | --- | --- |
| Category | Code | Quotation | 1 | 2 | 3 | 4 | 5 | 6 | 7 | 8 | 9 | 10 |
| Desire | active pursuit of accreditation |  | x |  |  |  |  |  |  |  |  |  |
|  | desire to be acknowledged | we are always studying, improving … and always ask many information about how to improve ourselves to be … acknowledged by other [parts] of the profession and other countries' (6) |  |  |  |  |  | x |  |  |  |  |
|  | feel good effect | the fact that this degree has been recognized by an international organization is a positive feeling (5) |  |  |  |  | x |  |  |  |  |  |
| Hierarchy | hierarchical nature | it has become more of a hierarchical endorsement exercise (5) | x |  |  |  | x |  |  |  |  |  |

| **Fear of Marginalisation** | | | | | | | | | | | | |
| --- | --- | --- | --- | --- | --- | --- | --- | --- | --- | --- | --- | --- |
| Category | Code | Quotation | 1 | 2 | 3 | 4 | 5 | 6 | 7 | 8 | 9 | 10 |
|  | not intrinsically beneficial, but has become normative | disadvantageous or not, we're still going to be doing it' (9)  'I think the realization will strike eventually that we don't really need to bend over backwards for it. (1) | x |  | x | x |  |  |  |  | x |  |
|  | not beneficial if highly performing | for higher[-ups] it's just a game …(9) |  |  |  |  |  |  |  |  | x |  |
|  | part of global community | you want to be part of the global community' (8) | x |  |  |  |  |  |  | x |  | x |
|  | regional collaboration | We'll try to work with other Asian countries to apply this international standard' (7) |  |  |  |  |  |  | x |  |  |  |

| **Political Pressures** | | | | | | | | | | | | |
| --- | --- | --- | --- | --- | --- | --- | --- | --- | --- | --- | --- | --- |
| Category | Code | Quotation | 1 | 2 | 3 | 4 | 5 | 6 | 7 | 8 | 9 | 10 |
| Pressure | political pressure for | The minister of education and the minister of health have tried … to push … to get the international standard (7) | x |  |  |  | x |  | x |  |  |  |
|  | political pressure against | there is a market for poor quality medical schools (5) |  | x |  |  | x |  |  |  |  |  |
| Funding | governmental funding | They will give the funding … for applying' (7) |  |  |  |  |  |  | x |  |  |  |
